# The Response of the Amputee Athlete Heart to Chronic Core Exercise: A Study on Hematological and Biochemical Parameters, and N-Terminal Pro Brain Natriuretic Peptide

**DOI:** 10.3390/medicina60050784

**Published:** 2024-05-09

**Authors:** Ahmet Kurtoğlu, Nurettin Konar, Faruk Akçınar, Madawi H. Alotaibi, Safaa M. Elkholi

**Affiliations:** 1 Department of Coaching Education, Faculty of Sport Science, Bandirma Onyedi Eylul University, 10200 Balikesir, Turkey; 2 Department of Physical Education and Sport Teaching, Faculty of Sport Sciences, Bandirma Onyedi Eylul University, 10200 Balikesir, Turkey; 3 Department of Coaching Education, Faculty of Sport Science, Inonu University, 44000 Malatya, Turkey; 4 Department of Rehabilitation Sciences, College of Health and Rehabilitation Sciences, Princess Nourah bint Abdulrahman University, P.O. Box 84428, Riyadh 11671, Saudi Arabia

**Keywords:** amputee soccer, biochemical parameters, NT-pro-BNP, hematological parameters, disability

## Abstract

*Background and Objectives*: mortality and morbidity due to cardiovascular causes are frequently experienced in amputees. Research on the effects of chronic exercise on biomarkers and cardiac damage indicators in these individuals is limited. The aim of this study was to investigate the effects of a core training program on brain natriuretic-related peptide, as well as hematological and biochemical parameters in amputee soccer players. *Materials and Methods*: The participants were randomly allocated to the following two groups: a core exercise group (CEG) and a control group (CG). While the CG continued routine soccer training, the CEG group was included in a core exercise program different from this group. During the study, routine hemogram parameters of the participants, various biochemical markers, and the concentration of brain natriuretic-related peptide (NT-pro-BNP) were analyzed. *Results*: after the training period, notable improvements in various hematological parameters were observed in both groups. In the CEG, there were significant enhancements in red blood cell count (RBC), hematocrit (HCT), mean corpuscular hemoglobin concentration (MCHC), and mean corpuscular hemoglobin (MCH) values. Similarly, the CG also showed substantial improvements in RBC, HCT, mean corpuscular volume (MCV), MCHC, MCH, red cell distribution width-standard deviation (RDW-SD), platelet-to-lymphocyte ratio (PLCR), mean platelet volume (MPV), and platelet distribution width (PDW). Moreover, in the CEG, serum triglycerides (TG) and maximal oxygen uptake (MaxVO_2_) exhibited significant increases. Conversely, TG levels decreased in the CG, while high-density lipoprotein (HDL), low-density lipoprotein (LDL), and MaxVO_2_ levels demonstrated substantial elevations. Notably, the N-terminal pro-brain natriuretic peptide (BNP) levels did not undergo significant changes in either the CEG or the CG following the core exercise program (*p* > 0.05). However, in the CEG, a meaningful positive correlation was observed between NT-pro-BNP and creatine kinase (CK) levels before and after the core exercise program. *Conclusions*: the findings emphasized the potential benefits of core training in enhancing specific physiological aspects, such as erythrocyte-related parameters and lipid metabolism, as well as aerobic capacity. Furthermore, the observed correlation between NT-pro-BNP and CK levels in the CEG provides intriguing insights into the unique physiological adaptations of amputee athletes.

## 1. Introduction

Limb amputation can cause problems due to a change in the center of gravity, impaired walking and running abilities, high energy expenditure, increased heart rate (HR), and decreased oxygen consumption [1]. These physiological and physical changes due to amputation necessitate in-depth investigations. In a recent study, it was concluded that individuals who underwent lower extremity amputation put more strain on their healthy feet and generally scored lower on more complex dynamic balance tests than did their normally developing peers [2]. This situation increases the risk of falls and injuries in amputees and causes deterioration of gait biomechanics. To prevent this situation, it has been argued that some specialized exercise practices should be performed according to the level of amputation and the effect it has created [3]. Core strengthening and stabilization exercises have strong potential for relieving common musculoskeletal disorders, chronic low back pain, muscle atrophy, loss of strength, and kinematic and mechanical disorders resulting from amputation [4]. A systematic meta-analysis by Fernández-Rodríguez et al. [5] suggested that core exercises can be an important method for relieving low back pain and disability. In addition, these exercises are effective in eliminating balance, proprioception, and functional disability by promoting hypertrophy of core muscles such as the transversus abdominus, lumbar multifidus [6], and gluteal and pelvic muscles [7]. In addition, chronic core exercises have been shown to have a positive impact on hematological parameters such as red blood cell count (RBC), hemoglobin (HGB), hematocrit (HCT), and mean corpuscular volume (MCV) [8] as well as biochemical parameters such as total cholesterol (TC), triglycerides (TG), high-density lipoprotein (HDL), and low-density lipoprotein (LDL) [9].

It is well known that the body’s metabolism changes during acute physical exertion. Cardiac damage indicators such as creatine kinase myocardial band (CK-MB) and brain natriuretic peptide (BNP) increase in response to acute exercise [10,11]. Similarly, indicators of muscle damage, such as creatine kinase (CK) and lactate dehydrogenase (LDH), increase [12]. After this damage, the levels of pro- and anti-inflammatory cytokines, such as interleukin-6 (IL-6), tumor necrosis factor (TNF-α), albumin, CRP and naturally occurring cytokine inhibitors and chemokines, increase [13]. Some physiological changes can be observed in amputees due to amputation. CK and LDH can be up to 300 times greater than normal in individuals who were amputated as a result of acute trauma [14]. Although these values decrease as a result of appropriate rehabilitation processes [15], the main reason is thought to be increased energy expenditure and fatigue as a result of amputation [16].

The effects of BNP in healthy people who participate in sports can be grouped as acute or chronic. In terms of acute effects, people who perform regular sports also have high NT-pro-BNP levels at rest after intense training. In terms of chronic effects, the NT-pro-BNP level in endurance athletes remains within the normal range at rest [17]. The increase in NT-pro-BNP after exercise depends on the duration and intensity of exercise [18,19]. In addition to the duration of exercise, age, gender, or disease state affect the NT-pro-BNP level [20]. The most important cause of postamputation mortality (39% survival rate at 7 years) in amputees is cardiac problems [21]; therefore, it is necessary to examine the physiological and morphological development of different types of exercise on the heart in these individuals. In our latest research, we have shown that the echocardiographic findings of amputee soccer players differ from those of nonamputee athletes and sedentary individuals [22]. This finding suggested that exercise has different morphological effects on the hearts of amputees.

In the literature, postoperative cardiac problems in amputees have been investigated, and research on the physiological effects of different types of exercise in these individuals is limited. Therefore, the aim of this study was to examine the acute and chronic effects of an eight-week core exercise program, NT-pro-BNP, and hematological and biochemical effects on amputee soccer players. In this context, the hypothesis of our research was that a “Chronic core exercise program has a positive effect on hematological and biochemical parameters and NT-pro BNP levels in amputee football players”.

## 2. Materials and Methods

### 2.1. Participants

To determine the number of participants included in the study, G Power (version 3.1.9.7) software was used (G Power: Dusseldorf University, Dusseldorf, Germany). When the type I error (α) was 0.05, the power (1 − β) was 0.80, the effect size was 1.7, and the actual power was 80.9 in the power analysis, it was determined that 5 participants in the exercise group (CEG) and the control group (CG) should be included in the study. According to the power analysis results, 7 participants in the CEG group and 6 participants in the CG group were included in the study. Personal interviews were conducted with the participants and team coaches who participated in the study; the purpose, potential risks, duration, and details of the study were explained. Volunteers signed an informed consent form. The amputee soccer team of Malatya city, comprising 13 active and 2 inactive athletes, was divided randomly into the following groups: CEG = 7, CG = 6. After the determination of the CEG and CG, a face-to-face meeting was held with the athletes, during which they were informed about the study schedule, their planning, the rules to be followed during the study, and the tests to be performed. Thirteen athletes participated in the first tests of the study. Participants who (a) had pain in the core region, (b) were taking medications that interfered with the results of the study, (c) had myocardial disease, (d) had thyroid-like disorders, (e) had hypertension, (f) had severe or moderate valvular heart disease, or (g) smoked were not considered. In this regard, 2 athletes (CEG = 1, CG = 1) who did not meet the study criteria were excluded from the study. The necessary approval for the study was obtained from the Malatya Clinical Research Ethics Committee (approval number: 2020/142). The study was conducted in accordance with the criteria of the Declaration of Helsinki. In addition, participants were asked to refrain from high-intensity exercise and substances such as alcohol and caffeine during the examination and during the performance of the tests. After the participants’ demographic data were recorded (Table 1), blood tests were performed.

### 2.2. Experimental Design of Study

**Figure 1:**
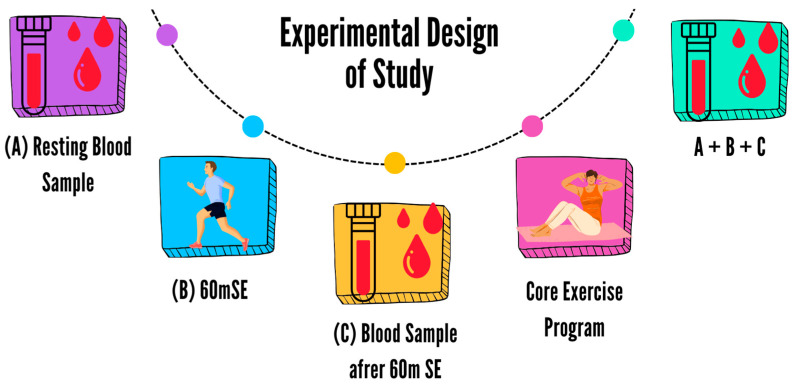
Experimental design of study: SE: submaximal exercise.

After the participants were randomly divided into the CEG and CG groups, both groups continued with soccer training, while CEG included extra core exercise training. Meanwhile, CG performed a warm-up with a ball. On the day of blood collection, the athletes received 8 h of sleep and were warned not to take stimulants on an empty stomach (A). After the first blood collection, a 60-min submaximal exercise (60 mSE) (10 min warm-up, 5 min stretching, 15 × 2 min 70–75% maximum HR (HRmax), 10 min jogging, 5 min stretching) was performed (B). The Karvonen formula was used to calculate the HR reserves of the participants before each test session [23]. The second blood sampling was performed immediately after the exercise (C). The same procedure was repeated at the end of the eighth week (A-B-C) (Figure 1).

### 2.3. Core Training Program

The 8-week core training program was incorporated and applied to the season training program. Before the normal training, all participants warmed up (10 min) and stretched (5 min), and the above training program was applied to the CEG group. After the program, the participants continued their normal soccer training (Table 2).

### 2.4. Data Collection

#### 2.4.1. Blood Tests

To determine the physiological responses to core training, the parameters MaxVO_2_, hematological parameters, NT-pro-BNP, CK, CK-MB, TG, LDL, HDL, and LDH were examined. For the hematological parameters and biochemical parameters, two 4-mL samples were collected from the participants. The collected blood samples were centrifuged at 2000 rpm for 10 min in a Hettich Rotofiz 32A centrifuge (Hettich, Tuttlingen, Germany) at room temperature of 22 to 25 degrees Celsius and the plasma was separated and analyzed via ELISA kits (Thermo Fisher Scientific, Waltham, MA, USA). Blood samples were collected from the subjects in 4 mm EDTA tubes and analyzed with a Sysmex XT-18.001 automatic blood count analyzer (Sysmex Corporation, Kobe, Japan). Biochemical parameters were analyzed using a Hitachi Cobas 6000 instrument (Roche Diagnostics, Rotkreuz, Switzerland).

#### 2.4.2. BMI Measurements of Participants

The BMI of the disabled population differed from that of the nondisabled population. For this reason, the BMIs of amputees were corrected according to the amputee coalition (AC). Based on the published sources and expert opinions, AC calculators corrected for proportions of total missing BM based on the following percentages: foot (Symes) = 1.30%, transtibial = 3.26%, transfemoral = 9.96%, and hip disarticulation/hemipelvectomy = 11.83%. This correction does not differ for men or women, unlike other estimates, which are slightly greater for women: 3.355% and 10.712% (transtibial and transfemoral, respectively). The estimated BM was calculated as follows:

Estimated BM = (BM without prosthesis)/(1.0 − AC% converted to decimal fraction) [24,25].

#### 2.4.3. Rockport 1 Mile Walking Test

This test was performed to determine the person’s cardiorespiratory fitness level. The person to be tested was instructed to walk as fast as possible. After the 1 mile was completed, the participant’s HR was measured with the help of the monitor. The 1 mile completion time was recorded.

MaxVO_2_ = 139.168 − (0.388 × age) − (0.077 × body weight) − (3265 × recorded walking time) − (0.156 × HR). Since the participants were male, +6.318 was added to the results [26,27]. 

### 2.5. Data Analysis

The SPSS package program 25 was used for the statistical procedures of the study. For the normality analysis of the data, the Shapiro–Wilk test was used. It was found that the data were not normally distributed, and nonparametric tests were preferred. In this context, Wilcoxon test was used to analyze the improvements between all parameters of CEG and CG at the end of eight weeks. Spearman’s correlation test was used to determine the relationships among the data obtained in the study. In addition, a repeated measures Anova test (2 groups × 2 times) was applied to examine the interaction of blood parameters between the groups after the core exercise program. Accordingly, the results were also analyzed in terms of time and group × time interaction. The effect size for this test was given by the partial eta squared (η_p_^2^) value. In this study, effect sizes were calculated according to Cohen’s d values. Accordingly, 0.2 indicated low, 0.5 indicated medium, and 0.8 indicated high effect sizes [28]. The level of significance in the study was determined to be 0.05.

## 3. Results

**Figure 2:**
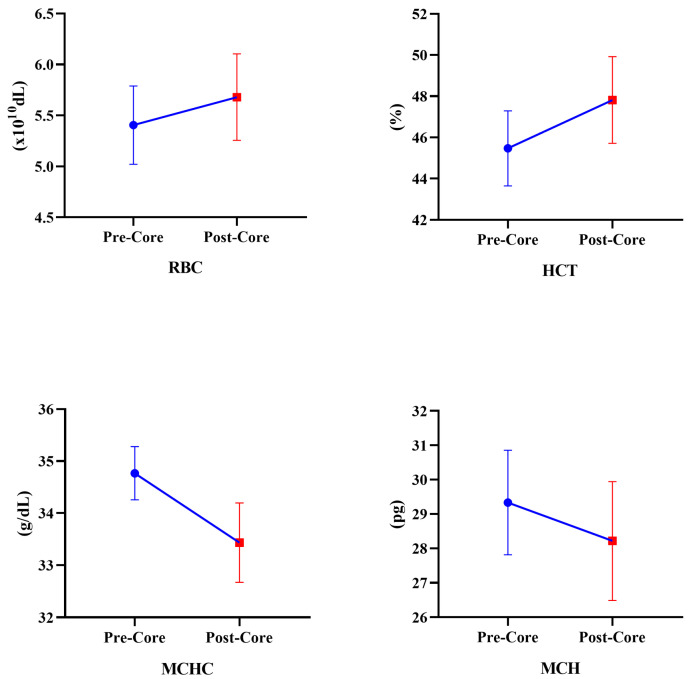
Changes in hematological parameters of CEG after core exercise program.

**Figure 3:**
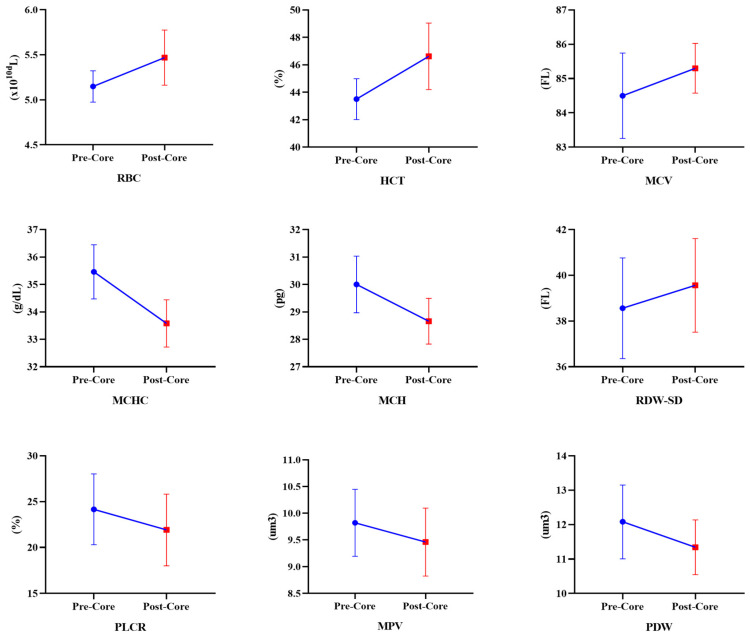
Changes in hematological parameters of CG after core exercise program.

Table 3 presents the chronic effects of the eight-week core exercise program on hematological parameters. According to these results, in the CEG, an increase in RBC (Z = −2.201, *p* = 0.028) and HCT (Z = −2.021, *p* = 0.028) values was observed. However, MCHC (Z = −2.027, *p* = 0.027) and MCH (Z = −2.226, *p* = 0.026) values decreased (Figure 2). In the CG, RBC (−2.203, *p* = 0.043), HCT (Z = −2.032, *p* = 0.042), MCV (Z = −2.023, *p* = 0.043), and RDW-SD (Z = −2.023, *p* = 0.043) values increased. On the other hand, MCHC (Z = −2.023, *p* = 0.043), MCH (Z = −2.023, *p* = 0.043), PLCR (Z = −2.023, *p* = 0.043), MPV (Z = −2.032, *p* = 0.042), and PDW (Z = −2.023, *p* = 0.043) values significantly decreased (Figure 3). According to the repeated measure Anova test results between CEG and CG, in the time interaction, the participants’ hematological parameters RBC [F_(1,9)_ = 33.714, η_p_^2^ = 0.78, *p* < 0.001], HCT [F_(1,9)_ = 36.569, η_p_^2^ = 0.80, *p* < 0.001], MCHC [F_(1,9)_ = 166.594, η_p_^2^ = 0.94, *p* < 0.001], MCH [F_(1,9)_ = 68.571, η_p_^2^ = 0.88, *p* < 0.001], PLCR [F_(1,9)_ = 7.628, η_p_^2^ = 0.45, *p* = 0.22], MPV [F_(1,9)_ = 5.829, η_p_^2^ = 0.39, *p* = 0.039], and PWD [F_(1,9)_ = 9.983, η_p_^2^ = 0.52, *p* = 0.012] parameters were significantly different. There was no significant difference in the group*time interaction (*p* > 0.05). When the hematological parameters were examined, the level of significance was greater in favor of CEG.

Table 4 presents the chronic effects of the eight-week core exercise program on biochemical parameters. According to these results, in the CEG, TG levels significantly increased after eight weeks (Z = −1992, *p* = 0.046). In contrast, in the CG, TG levels significantly decreased (Z = −2.023, *p* = 0.043). Moreover, HDL (Z = −2.023, *p* = 0.043) and LDL (Z = −2.023, *p* = 0.043) levels significantly increased. The MaxVO_2_ value significantly increased in the CEG (Z = −2.201, *p* = 0.028). MaxVO_2_ also significantly increased in the CG (Z = −2.023, *p* = 0.043). According to the repeated measure Anova test results between CEG and CG, there was a significant difference between the participants’ biochemical and physiological parameters HDL [F_(1,9)_ = 6.383, η_p_^2^ = 0.78, *p* = 032], LDL [F_(1,9)_ = 36.569, η_p_^2^ = 0.80, *p* < 0.001], and MaxVO_2_ [F_(1,9)_ = 21.413, η_p_^2^ = 0.80, *p* = 0.001] in time interaction. In the Group × Time interaction, participants’ TG [F_(1,9)_ = 16.212, η_p_^2^ = 0.64, *p* = 0.003] levels were significantly different. However, the amount of increase was greater in favor of CEG.

**Figure 4:**
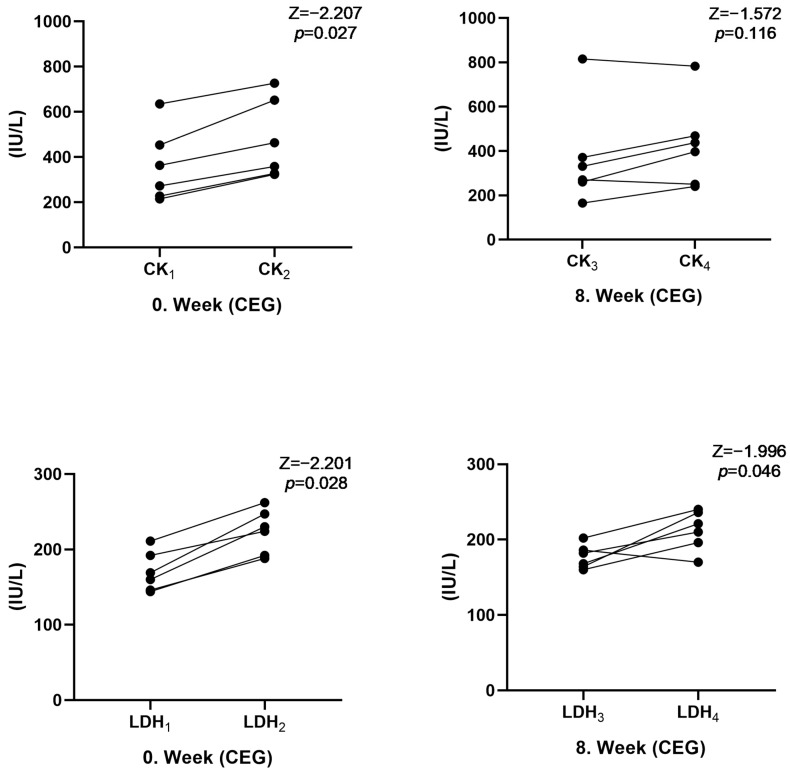
Acute effect of biochemical parameters on CEG: CK1: Resting CK result at week 0, CK2: week 0 CK level after 60 mSE, CK3: Resting CK result at week 8, CK4: 8th week CK level after 60 mSE.

Figure 4 shows the acute changes in the biochemical parameters of the participants. Accordingly, CK (Z = −2.207, *p* = 0.027) and LDH (Z = −2.201, *p* = 0.028) values increased significantly after 60 min of submaximal exercise at week 0 in the CEG. No changes were observed in these parameters at week 8. There was no change in other hematological or biochemical parameters in the CEG (*p* > 0.05). In the CG, no acute changes were observed in any of the hematological or biochemical parameters after 60 min of submaximal exercise (*p* > 0.05).

**Figure 5:**
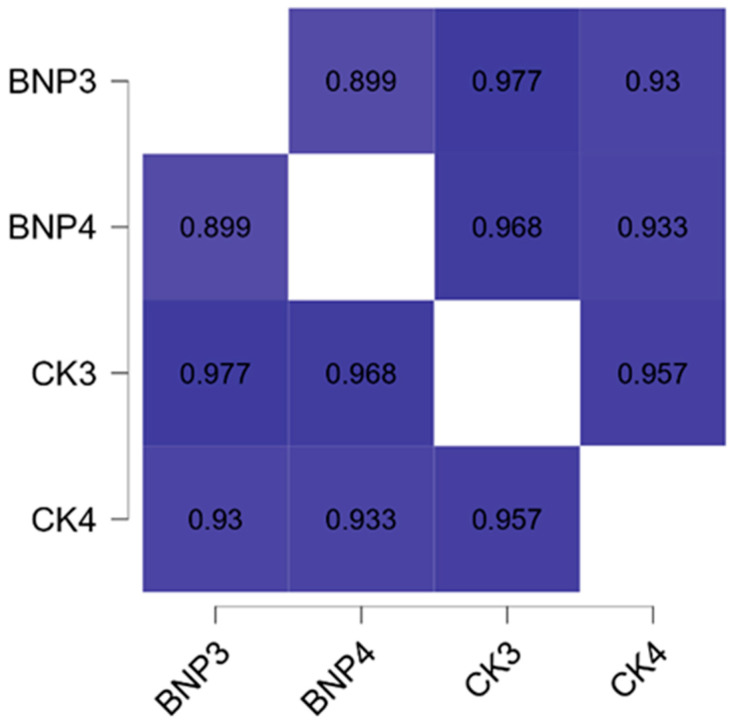
Pearson’s heatmap of CK and NT-pro-BNP at week eight: BNP3: NT-pro-BNP level before 60 mSE at the 8th week, BNP4: NT-pro-BNP level after 60 mSE at the 8th week, CK3: CK level before 60 mSE at the 8th week, CK4: CK level after 60 mSE at the 8th week.

Figure 5 shows the relationship between CK and NT-pro-BNP at week eight. Accordingly, a positive correlation was found between resting NT-pro-BNP and CK (r = 0.977, *p* < 0.001) and between NT-pro-BNP and CK after 60 mSE (r = 0.933, *p* = 0.007).

## 4. Discussion

Our study investigated the acute and chronic effects of an eight-week core exercise program on hematological and biochemical parameters in amputee soccer players. According to the results, RBC and HCT increased in the CEG. MCH and MCHC values decreased. Although similar improvements were observed in the CG, the positive changes were greater in favor of the CEG. Additionally, in the CEG, there was an increase in triglyceride levels, while the CG levels decreased. The CG also exhibited an increase in HDL and LDL. In the CEG, after 60 mSE at week 0, there was an increase in CK and LDH levels, which was not observed at week 8. In this regard, our initial hypothesis that ‘Eight-week core exercise positively affects hematological and biochemical parameters in amputee soccer players’ was confirmed.

Hematological parameters respond differently to acute and chronic exercise. During acute exercise, the amount of circulating erythrocytes, the circulation velocity, the amount of oxygen delivered to the active muscle, and the metabolic rate increase as a function of the intensity of the activity [29]. Belviranli et al. [30] analyzed hematological parameters after acute high-intensity exercise (HIIT). According to the results of this study, it was concluded that HCT, HGB, RBC, MCH, and PLT values increased significantly after HIIT, and returned to their resting levels 3 h after the end of exercise [30]. Bashafaat et al. [31] concluded that interval and blood flow restricted exercises caused an increase in WBC, RBC, HGB, HCT, and PLT values in amateur cyclists. In a study by Azarbayjani et al. [32] in which the effects of aerobic and anaerobic exercise on hematological parameters were examined, it was concluded that aerobic exercise had a less acute effect on hematological parameters. In this context, exercise-induced hematological changes appear to depend on the type, intensity, and duration of exercise [29]. According to the literature, aerobic exercise generally does not cause significant changes in hematological parameters. In addition, high-intensity exercise significantly changes hematological parameters. In a study conducted by Atan and Alacam [33], it was reported that hematological parameters increased 1 min after acute aerobic exercise but returned to resting levels after 60 min. Considering these results, the absence of hematological changes in CEG after acute exercise, the low intensity of 60 mSE performed with 70 to 75% HR, and blood samples taken longer than 1 min after exercise can be shown in our study. As a result of chronic exercise, the oxygen carrying capacity increases. In this case, an increase in hematological parameters such as blood RBC and HGB was observed [34]. Chronic exercise may have different effects on hematological parameters. In a study conducted by Bobeuf et al. [35], the effects of six months of resistance exercise on hematological parameters in elderly women and men were examined, and it was determined that there was no significant change in these parameters after six months. Since muscle atrophy in elderly individuals [36] reduces the amount of oxygenation of the muscles [37], it is common that there is no change in hematological parameters. In our study, RBC and HCT increased in the CEG. This indicated that the amount of oxygen entering circulation increased after the core exercise program. The acute fatigue observed in CK and LDH in the first week did not occur in the last week, as metabolic adaptation to training with an increase in RBC and HCT caused the intake of metabolites such as lactate released from skeletal muscles during high-intensity exercise [38]. These results support each other, and it can be concluded that core exercises also reduced fatigue by increasing oxygenation. In our study, reduction in erythrocyte parameters, such as MCH and MCHC, were also detected. As a result of high-intensity exercise, some reduction in RBC and HCT was observed, and this is defined as anemia in athletes [39]. In one study, a decrease in hematological parameters was observed in young soldiers who walked 35 km per day for six days. An early (two days) improvement was observed in RBC, HCT and MCV counts. HGB was characterized by a persistent (more than four days) reduction in MCH and MCHC [40]. In this case, it is thought that the decreases in MCH and MCHC in both groups (more in CEG) were due to the decrease in hematological parameters resulting from athlete’s anemia.

It has been reported that exercise has positive effects on fat and carbohydrate metabolism [41]. In general, significant changes in obesity-related parameters, such as improvements in lipid profiles; decreases in triglyceride, cholesterol, and LDL levels; increases in HDL levels; and decreases in body fat mass and body mass index, are more evident with regular and continuous exercise programs [42,43]. Kokkinos and Fernhall [44] examined the blood lipoprotein levels of athletes during preseason training and reported a significant decrease in triglyceride levels, with no significant difference in HDL or LDL cholesterol levels. The amount of triglycerides increased due to the training program in the CEG. From the oxidation of fatty acids released after the breakdown of triglycerides in cells, 3.5 times more energy per mole was obtained [45]. We hypothesized that most of the increased energy requirements of the muscles during exercise in the CEG can be met by the oxidation of triglycerides that pass into the blood from fat stores.

The literature shows that physical activity reduces the risk of coronary heart disease [46]. In this context, cardiac markers play an important role in understanding the intensity of exercise. Some academic studies have shown that although there is an increase in cardiac damage markers such as cardiac troponin, CK-MB, and NT-pro-BNP after moderate walking and low and moderate exercise intensity, this increase is not significant [47,48,49]. However, biochemical levels such as NT-pro-BNP, troponin, and CK-MB have been found to be elevated in long-distance marathon runners [19,50,51]. According to the literature, the intensity of exercise is directly proportional to the increase in cardiac parameters. An increase in cardiac markers was detected, especially during high-intensity exercise programs. Since the exercise program used in our study was not a high-intensity program, results similar to those found in the studies reviewed in the literature were found.

There are two main explanations for muscle damage caused by exercise. The first is unaccustomed stress, and the second is metabolic and chemical processes associated with tissue damage. Muscle damage is caused by structural deterioration, especially tearing in the Z-band of myofibrils. The different Z-band thicknesses of different muscle fiber types could explain the different degrees of damage caused by the same exercise in different fiber types [52]. This muscle damage usually leads to an increase in the levels of some enzymes, such as CK and LDH. Age, gender, and type and intensity of exercise affect the magnitude of the increase in these parameters [53]. In studies examining markers of muscle damage such as CK and LDH acutely and chronically, it was concluded that although CK and LDH parameters increased by 300% during the acute effects of exercise, the increase in these parameters decreased when exercise was adopted [54,55]. The extent of cardiorespiratory and motor adaptation to training depends on the intensity, duration, and frequency of training. For this harmony to reach the desired level, a training duration of 8 to 12 weeks is recommended [56].

One of the important findings in our study was that a positive correlation was detected between NT-pro-BNP and CK in the CEG at week eight. Studies have reported that NT-pro-BNP does not increase immediately in those who exercise [11]. During acute exercise, the working capacity of skeletal and cardiac muscles increases due to increased physical activity [57]. The levels of biochemical parameters such as CK and CK-MB also increased in this case. Muscles that adapted to chronic exercise were less fatigued, and therefore, less CK and CK-MB increases were observed [58]. In our study, after the eight-week core exercise program, an increase in CK and NT-pro-BNP levels was observed, although this increase was not significant in the CEG. According to the literature, it can be concluded that the excessive training load applied to amputees causes fatigue in the heart and skeletal muscles. In our previous studies, it was concluded that the heart morphology of amputees is different from that of healthy individuals [22]. In this research, the sample consisted of amputee soccer players. Due to the limited ability to perform sufficient classification, these individuals were not classified based on the level of amputation. However, a detailed investigation of the effects of amputation on NT-pro-BNP, hematological and biochemical parameters could be explored in future studies. By categorizing participants based on their level of amputation, it may be possible to analyze the changes in biochemical parameters more comprehensively. In this study, biochemical parameters such as lipid parameters and indicators of muscle and heart damage, such as CK and CK-MB, were analyzed. Nevertheless, more comprehensive studies examining certain cytokines, liver enzymes, and hormones may provide more realistic results. Notably, this research focused solely on the hematological and biochemical effects of core exercises. Future studies could investigate the physiological mechanisms in amputee individuals by combining different exercise programs that involve both aerobic and anaerobic components. By implementing various exercise programs that encompass a range of aerobic and anaerobic characteristics, significant insights into the physiological responses of amputee individuals may be gained. Such research could help expand our understanding of the broader effects of exercise on the hematological, biochemical, and physiological aspects of amputee soccer players.

## 5. Conclusions

In our study, core exercise for eight weeks resulted in greater changes in some hematological parameters in the CEG. However, no positive effect of core exercises on biochemical parameters was observed. There was a difference between the BMIs of the CEG and CG, although no significant difference was observed. This explains the decrease in TG and the increase in HDL and LDL in the two CGs. Another important result of this study was that core exercises can increase the oxygen requirement of the muscles in amputees without creating stress on the left ventricle and myocardium. These findings contribute to the existing knowledge on exercise adaptations in this unique population and may inform the development of tailored training strategies to optimize athletic performance and overall well-being in amputee soccer players. However, further research is warranted to comprehensively understand the mechanisms driving these responses and to explore the potential long-term effects of core exercise on overall health and sports performance in this population. In conclusion, the results of this research suggest that differences in the physiological structures of amputees should be examined in depth. In addition, the positive correlation between NT-pro-BNP and CK at the end of eight weeks was suggestive. It is recommended to examine the effect of acute and chronic exercise at different exercise intensities.

## Figures and Tables

**Table 1:** Identifiable information of participants (Mean = x, Standard Deviation = SD).

Parameter	CEG (n = 6) x ± SD	CG (n = 5) x ± SD	*p*
Age (year)	23.8 ± 4.2	28.3 ± 6.6	0.196
Height (cm)	174.4 ± 11.5	175.1 ± 9.6	0.907
Weight (kg)	60.6 ± 5.7	72.8 ± 16	0.161
BMI (kg/m^2^)	20.2 ± 3.9	23.5 ± 4.1	0.211

BMI: Body Mass Index, CEG: Core Exercise Group, CG: Control Group.

**Table 2:** Core training program.

Weeks	1. Day	2. Day	3. Day
1. Week	Bicycle Crunch (10 × 3 sets) Reverse Crunch (10 × 3 sets) Bird Dog (10 × 3 sets) Reverse Pendulum (10 × 3 sets)	Prone Plank (3 × 15 s) Stability Ball Plank (3 × 15 s) Right Side Bridge (3 × 15 s) Left Side Bridge (3 × 15 s) Back Plank (3 × 15 s)	Russian Twist (10 × 3 sets) Flutter Kick (10 × 3 sets) Side Double-Leg Lift (10 × 3 sets) Swimmer (10 × 3 sets)
2. Week	Superman (15 × 3 sets) Leg Lower (15 × 3 sets) Side to Side Twist (15 × 3 sets) Dumbbell Side Bend (15 × 3 sets)	Ball Rotating Crunch (3 × 20 s) Ball Supine Bridge (3 × 20 s) Ball Hyperextension (3 × 20 s) Ball Crunch (3 × 20 s)	Sit-Up (15 × 3 sets) Right Side Lateral Raise (15 × 3 sets) Left Side Lateral Raise (15 × 3 sets) Press-up (15 × 3 sets)
3. Week	Open-Book Rib Cage (15 × 3 sets) Hanging Knee Raise (15 × 3 sets) Three Way Hanging Knee Raise (15 × 3 sets) Static Back Extension (15 × 3 sets)	Superman With Medicine Ball (3 × 20 s) Diagonal Medicine Ball Chop (15 × 3 sets) Medicine Ball Pullover Pass (15 × 3 sets) Medicine Ball Overhead Throw (15 × 3 sets)	Plate V-Up (15 × 3 sets) Jackknife (15 × 3 sets) Sit-Up (15 × 3 sets) Back Extension (15 × 3 sets)
4. Week	Assisted Squat (10 × 3 sets) Side to Side Twist (20 × 3 sets) Push-Up (10 × 3 sets) Slide-Board Thrust (15 × 3 sets)	Waiting Back Extension (3 × 30 s) Waiting Toe Tabs (3 × 30 s) Waiting Leg Lower (3 × 30 s) Waiting Leg Lower with Seated Rotation (3 × 30 s)	Vertical Leg Crunch (20 × 3 sets) Vertical Leg Rotation (20 × 3 sets) Dumbbell Side Bend (20 × 3 sets) Glute Ham Raise (20 × 3 sets)
5. Week	Bicycle Crunch (20 × 3 sets) Reverse Crunch (20 × 3 sets) Bird Dog (20 × 3 sets) Reverse Pendulum (20 × 3 sets)	Prone Plank (3 × 30 s) Stability Ball Plank (3 × 30 s) Right Side Bridge (3 × 30 s) Left Side Bridge (3 × 30 s) Back Plank (3 × 30 s)	Russian Twist (20 × 3 sets) Flutter Kick (20 × 3 sets) Side Double-Leg Lift (20 × 3 sets) Swimmer (20 × 3 sets)
6. Week	Superman (20 × 3 sets) Leg Lower (20 × 3 sets) Side to Side Twist (20 × 3 sets) Dumbbell Side Bend (20 × 3 sets)	Ball Rotating Crunch (3 × 30 s) Ball Supine Bridge (3 × 30 s) Ball Hyperextension (3 × 30 s) Ball Crunch (3 × 30 s) Ball Plank (3 × 30 s)	Sit-Up (20 × 3 sets) Right Side Lateral Raise (20 × 3 sets) Left Side Lateral Raise (20 × 3 sets) Press-up (20 × 3 sets)
7. Week	Open-Book Rib Cage (20 × 3 sets) Hanging Knee Raise (20 × 3 sets) Three Way Hanging Knee Raise (20 × 3 sets) Static Back Extension (20 × 3 sets)	Superman With Medicine Ball (3 × 30 s) Diagonal Medicine Ball Chop (20 × 3 sets) Medicine Ball Pullover Pass (20 × 3 sets) Medicine Ball Overhead Throw (20 × 3 sets)	Plate V-Up (20 × 3 sets) Jackknife (20 × 3 sets) Sit-Up (20 × 3 sets) Back Extension (20 × 3 sets)
8. Week	Assisted Squat (15 × 3 sets) Side to Side Twist (25 × 3 sets) Push-Up (15 × 3 sets) Slide-Board Thrust (20 × 3 sets)	Waiting Back Extension (3 × 40 s) Waiting Toe Tabs (3 × 40 s) Waiting Leg Lower (3 × 40 s) Waiting Leg Lower with Seated Rotation (3 × 40 s)	Vertical Leg Crunch (25 × 3 sets) Vertical Leg Rotation (25 × 3 sets) Dumbbell Side Bend (25 × 3 sets) Glute Ham Raise (25 × 3 sets)

**Table 3:** Chronic effect of core exercise program on hematological parameters.

Parameters	Group	Pre-Core Exercise	Post-Core Exercise	Z	*p*	Time Interaction η_p_^2^/F/*p*	Group × Time Interaction η_p_^2^/F/*p*
WBC (×10^7^ L)	CEG CG	7.6 ± 2.0 8.9 ± 2.7	7.7 ± 1.5 7.7 ± 1.3	−0.314 −1.214	0.753 0.225	0.04 0.437 0.525	0.05 0.474 0.509
RBC (×10^10^ dL)	CEG CG	5.4 ± 0.3 5.1 ± 0.1	5.6 ± 0.4 5.4 ± 0.3	−2.201 −2.023	0.028 0.043	0.78 33.714 <0.001	0.02 0.193 0.671
HGB (g/dL)	CEG CG	15.8 ± 0.6 15.4 ± 0.9	15.9 ± 0.8 15.6 ± 1.1	−0.944 −0.405	0.345 0.686	0.14 1.479 0.255	0.00 0.048 0.831
HCT (%)	CEG CG	45.4 ± 1.8 43.5 ± 1.4	47.8 ± 2.1 46.6 ± 2.4	−2.201 −2.032	0.028 0.042	0.80 36.569 <0.001	0.07 0.725 0.417
MCV (FL)	CEG CG	84.3 ± 3.7 78.5 ± 14.1	84.4 ± 4.5 85.0 ± 0.7	−0.949 −2.023	0.343 0.043	0.07 0.748 0.410	0.04 0.452 0.518
MCHC (g/dL)	CEG CG	34.7 ± 0.5 35.4 ± 0.9	33.4 ± 0.7 33.5 ± 0.8	−2.207 −2.023	0.027 0.043	0.94 166.594 <0.001	0.34 4.822 0.056
MCH (pg)	CEG CG	29.3 ± 1.52 30.0 ± 1.0	28.2 ± 1.7 28.6 ± 0.8	−2.226 −2.023	0.026 0.043	0.88 68.571 <0.001	0.05 0.567 0.471
PLT (×10^7^ dL)	CEG CG	210.3 ± 61.8 239.2 ± 70.1	223.5 ± 69.5 233.2 ± 65.3	−0.943 −0.944	0.345 0.345	0.02 0.223 0.648	0.15 1.596 0.238
RDW-SD (FL)	CEG CG	40.1 ± 2.8 38.5 ± 2.2	40.0 ± 3.0 39.5 ± 2.0	−0.943 −2.023	0.345 0.043	0.02 1.188 0.644	0.04 1.552 0.599
RDW-CV (%)	CEG CG	13.3 ± 0.7 12.8 ± 0.8	13.1 ± 0.7 12.9 ± 0.7	−0.946 −0.962	0.344 0.336	0.00 0.009 0.854	0.06 0.140 0.468
PLCR (%)	CEG CG	24.6 ± 6.2 24.6 ± 6.2	23.1 ± 7.4 21.9 ± 3.8	−1.363 −2.023	0.173 0.043	0.45 7.628 0.022	0.02 0.239 0.637
MPV (μm^3^)	CEG CG	9.9 ± 0.9 9.8 ± 0.6	9.7 ± 1.2 9.4 ± 0.6	−0.944 −2.032	0.345 0.042	0.39 5.829 0.039	0.03 0.360 0.563
PDW (μm^3^)	CEG CG	12.2 ± 2.0 12.0 ± 1.0	11.5 ± 2.0 11.3 ± 0.7	−1.472 −2.023	0.141 0.043	0.52 9.983 0.012	0.00 0.027 0.873
PCT (%)	CEG CG	0.2 ± 0.0 0.2 ± 0.0	0.2 ± 0.0 0.2 ± 0.0	−0.863 −0.962	0.388 0.336	0.00 0.057 0.816	0.16 1.761 0.217

WBC: White Blood Cells, RBC: Red Blood Cells, HGB: Hemoglobin, HCT: Hematocrit, MVC: Mean Corpuscular Volume, MCHC: Mean Erythrocyte Hemoglobin Concentration, MCH: Mean Erythrocyte Hemoglobin, PLT: Platelets, RDW-SD: Red Cell Distribution Width—Standard Deviation, RDW-CV: Red Cell Distribution Width—Coefficient of Variation, PLCR: Platelet Larger Cell Ratio, MPV: Mean Platelet Volume, PDW: Platelet Distribution Width, PCT: Plateletcrit.

**Table 4:** Chronic effect of core exercise program on biochemical and physiological parameters.

Parameters	Group	Pre-Core Exercise	Post-Core Exercise	Z	*p*	Time Interaction η_p_^2^/F/*p*	Group × Time Interaction η_p_^2^/F/*p*
TG (mg/dL)	CEG CG	109.7 ± 56.4 184.1 ± 72.3	161.5 ± 84.4 108.3 ± 45.8	−1.992 −2.023	0.046 0.043	0.06 0.574 0.468	0.64 16.212 0.003
HDL (mg/dL)	CEG CG	49.4 ± 17.8 46.3 ± 10.0	51.6 ± 22.9 50.9 ± 11.1	−0.736 −2.023	0.462 0.043	0.41 6.383 0.032	0.08 0.817 0.390
LDL (mg/dL)	CEG CG	67.1 ± 19.1 60.0 ± 26.3	72.0 ± 23.6 78.8 ± 15.0	−1.153 −2.023	0.249 0.043	0.57 12.289 0.007	0.32 4.292 0.068
CK (IU/L)	CEG CG	361.5 ± 161.5 232.0 ± 151.2	368.6 ± 229.6 168.8 ± 56.7	−0.105 −0.674	0.917 0.500	0.01 0.105 0.753	0.01 0.166 0.693
CK-MB (IU/L)	CEG CG	16.9 ± 9.2 13.9 ± 3.6	13.5 ± 6.0 11.5 ± 3.9	−1.153 −1.214	0.249 0.225	0.33 4.518 0.062	0.01 0.160 0.752
LDH (IU/L)	CEG CG	170.3 ± 26.5 165.0 ± 5.5	177.0 ± 15.9 170.8 ± 22.1	−1.153 −0.405	0.249 0.686	0.12 1.228 0.297	0.00 0.006 0.940
MaxVO_2_	CEG CG	14.7 ± 4.8 14.7 ± 7.3	21.1 ± 8.1 19.8 ± 6.7	−2.201 −2.023	0.028 0.043	0.70 21.413 0.001	0.02 0.253 0.627
NT-pro-BNP (ng/L)	CEG CG	537.0 ± 208.3 749.5 ± 483.3	549.4 ± 199.3 733.3 ± 459.0	−0.734 −0.135	0.463 0.893	0.00 0.004 0.951	0.02 0.230 0.643

TG: Triglyceride, HDL: High-Density Lipoprotein, LDL: Low-Density Lipoprotein, CK: Creatine Kinase, CK-MB: Creatine Kinase-MB, LDH: Lactate Dehydrogenase, MaxVO_2_: Maximal Oxygen Uptake, NT-pro-BNP: N-terminal pro-B-type natriuretic peptide.

## Data Availability

Data supporting the findings of this study are available from the corresponding author upon reasonable request.
